# Subcutaneous and mediastinal emphysema after lobectomy: Two case reports with different courses

**DOI:** 10.1016/j.ijscr.2025.111689

**Published:** 2025-07-16

**Authors:** Yuki Shindo, Hiroya Ishihara, Takuro Morita, Kiyoshi Sato, Nobuharu Hanaoka, Takahiro Katsumata

**Affiliations:** a Department of Thoracic Surgery, Takatsuki Red Cross Hospital, Takatsuki, Japan; b Department of Thoracic Surgery, Kishiwada City Hospital, Kishiwada, Japan; c Department of Thoracic Surgery, Yao Tokushukai General Hospital, Yao, Japan; d Department of Thoracic and Cardiovascular Surgery, Osaka Medical and Pharmaceutical University, Takatsuki, Japan

**Keywords:** Subcutaneous emphysema, Mediastinal emphysema, Pneumomediastinum, Pneumothorax, Postoperative complications, Case report

## Abstract

**Introduction:**

Subcutaneous and mediastinal emphysema after lobectomy is uncommon and is typically caused by minor air leakage from the residual lung; however, the development of a contralateral pneumothorax is particularly rare, and if missed, it can be fatal.

**Presentation of cases:**

Two cases of subcutaneous and mediastinal emphysema occurring after a lobectomy in a 77-year-old man and a 78-year-old man are reported here. The former received conservative treatment, while the latter he developed a pneumothorax on the side contralateral to the surgical site after readmission. Chest tube drainage was then performed.

**Discussion:**

The occurrence of subcutaneous and mediastinal emphysema after lobectomy is not uncommon. Conservative management is often sufficient if no pneumothorax is present, as in Case 1. However, in Case 2, pneumothorax occurred on the contralateral side due to a minor air leakage, which is an unusual complication. Generally, a pneumothorax of the residual lung on the surgical side occurs earlier than a pneumothorax on the contralateral lung. The earlier onset on the contralateral side in Case 2 suggests that the pressure threshold for rupturing the contralateral mediastinal pleura and/or alveoli may have been lower than that for collapsing the residual lung on the operative side. We recommend that pulmonologists should also monitor the contralateral side and that follow-up radiographic examinations should be performed a few hours later.

**Conclusion:**

Pulmonologists should monitor the side contralateral to the surgical site, even if the emphysema is caused by a postoperative minor air leakage.

## Highlights

• Two postoperative subcutaneous emphysema cases after lobectomy; one uniquely developed a contralateral pneumothorax.
• Case 1 was managed conservatively, while Case 2 required urgent chest drainage four hours after presentation.
• This is the first reported case of contralateral pneumothorax secondary to postoperative subcutaneous emphysema.
• Highlights the importance of monitoring the contralateral hemithorax, even in the absence of ipsilateral pneumothorax.

## 1 Introduction

Subcutaneous and mediastinal emphysema after lobectomy is not uncommon and is typically attributed to minor air leakage from the residual lung. However, such cases have not been reported yet. Treatment usually involves conservative management (Case 1) [1], with chest tube drainage is performed if a pneumothorax occurs on the surgical side. Herein, we describe a case in which a pneumothorax developed on the contralateral side following the onset of subcutaneous and mediastinal emphysema (Case 2). This case report has been reported in line with the SCARE 2025 criteria [2].

## 2 Presentation of cases

### 2.1 Case 1

**Fig. 1:**
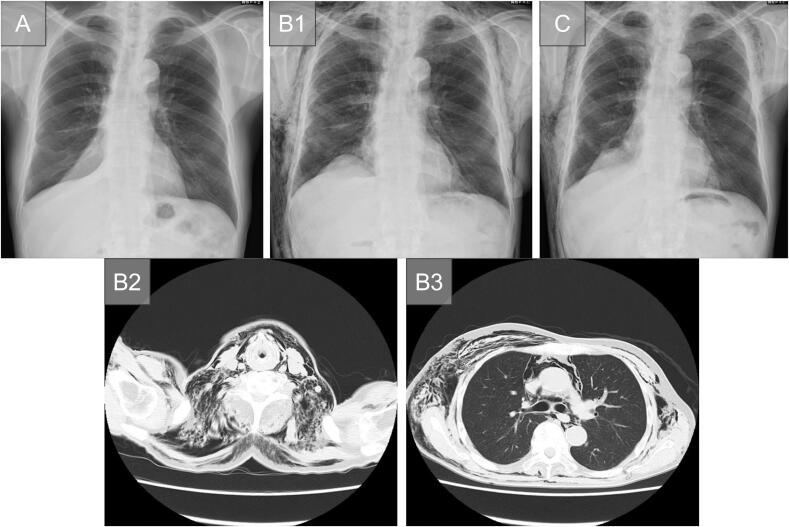
(Case 1) (A) Chest X-ray shows no subcutaneous emphysema prior to discharge (POD6). (B) (B1) Chest X-ray at readmission shows subcutaneous and mediastinal emphysema (POD7). Note the linear image of the pneumomediastinum along the left contour of the heart. Chest CT at admission shows subcutaneous and mediastinal emphysema from the neck (B2) to the chest (B3) (POD7). No obvious pneumothorax was observed. (C) Chest X-ray shows a reduction in subcutaneous emphysema compared to that observed prior to discharge (POD11). Abbreviations: CT: computed tomography; POD: postoperative day.

A 77-year-old man was diagnosed with primary lung adenocarcinoma in the right lower lobe (cT1cN0M0, Stage IA3), and surgical treatment was planned. He was intubated easy: first-pass, Cormack-Lehane I, with a left-sided double-lumen tube (Endo-Bronch® 37 Fr, Parker Medical, Highlands Ranch, CO, USA) under fiberoptic bronchoscopy. A standard Video-assisted thoracic surgery (VATS) right lower lobectomy was performed using the endostapler. A water-seal test performed before chest closure demonstrated an air leak originating from the lung parenchyma, whereas no leakage was detected at the bronchial stump. Inferior mediastinal lymph-node dissection was performed, but the contralateral bronchus could not be visualized. A clamp test was performed on postoperative day 3 (POD3), and because an upright X-ray on POD4 was unremarkable, the drain was removed that day; he was discharged on POD6 (Fig. 1A). However, on POD7, he returned with head-to-neck subcutaneous emphysema; exam revealed neck/back crepitus, SpO₂ 97 % on room air, clear lung fields, and normal labs. Chest X-ray and computed tomography (CT) confirmed the diagnosis of subcutaneous and mediastinal emphysema without pneumothorax (Fig. 1B1-3). He was readmitted the same day and managed conservatively with a chest band. On POD11 (4 days after his readmission), his chest X-ray showed a reduction in the subcutaneous emphysema (Fig. 1C). He was then discharged on POD12.

### 2.2 Case 2

**Fig. 2:**
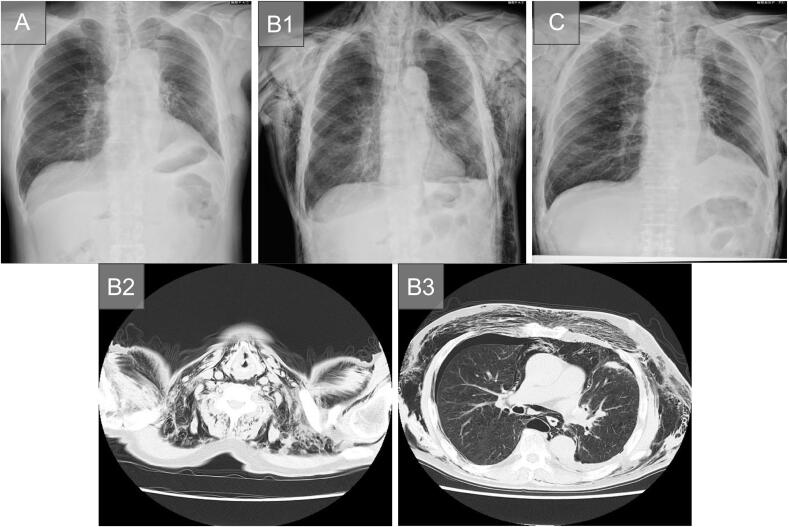
(Case 2) (A) Chest X-ray shows no subcutaneous emphysema prior to discharge (POD6). (B1) Chest X-ray at readmission shows subcutaneous and mediastinal emphysema (POD7). A slight suspicion of right pneumothorax. Chest CT at readmission shows subcutaneous and mediastinal emphysema from the neck (B2) to the chest (B3) (POD7). A slight suspicion of right pneumothorax. (C) Chest X-ray shows a reduction in subcutaneous emphysema compared to that observed prior to discharge (POD22). Abbreviations: CT: computed tomography; POD: postoperative day.

A 78-year-old man was diagnosed with primary lung adenocarcinoma in the left upper lobe (cT1bN0M0, Stage IA2), and surgical treatment was planned. He was intubated easy, as in Case 1. A standard VATS left upper lobectomy was performed using the endostapler. A water-seal test performed before chest closure revealed no air leak from either the lung parenchyma or the bronchial stump. Superior mediastinal lymph-node dissection was performed, but the contralateral bronchus could not be visualized. A clamp test was performed on POD2, and because an upright X-ray on POD4 was unremarkable, the drain was removed that day; he was discharged on POD6 (Fig. 2A). However, on POD7, he returned with head-to-neck subcutaneous emphysema; exam revealed neck/back crepitus, SpO₂ 97 % on room air, clear lung fields, and normal labs.

**Fig. 3:**
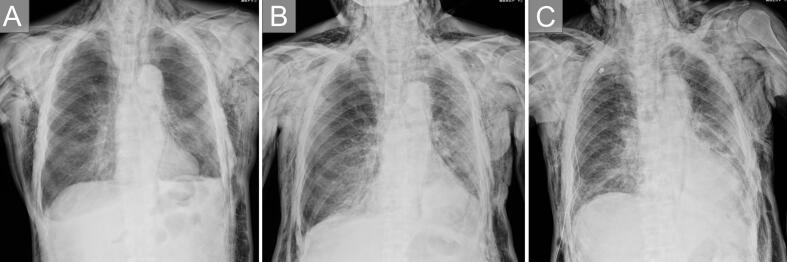
(A) Subcutaneous and mediastinal emphysema seen on the chest X-ray at readmission. A slight suspicion of right pneumothorax. (B) Chest X-ray 4 h later showing progression of the right pneumothorax. (C) Right lung expansion is observed after chest drainage.

Chest X-ray and CT confirmed the diagnosis of subcutaneous and mediastinal emphysema, and a slight pneumothorax was noted on the opposite side of the surgical side (Fig. 2B1-3). At that time, there was no sufficient space to place a chest tube drain. The patient was then urgently admitted on the same day. Upon admission, no pneumothorax was observed on the surgical side (Fig. 3A). However, due to the enlargement of the subcutaneous emphysema, a chest X-ray was performed at 4 h post-admission, revealing progression of contralateral pneumothorax (Fig. 3B). Chest tube drainage was then performed (Fig. 3C). After tube placement, air leakage was noted, but it resolved by POD8 (2 days after readmission). A clamp test was performed on POD10, and the chest tube was removed on POD15 (9 days after readmission). The patient was discharged on POD22 (Fig. 2C).

## 3 Discussion

The presenting complaints of patients with subcutaneous and mediastinal emphysema generally include central chest pain radiating to the back, new-onset rhinolalia, and shortness of breath [3].

The mechanism for subcutaneous emphysema and spontaneous pneumomediastinum (SPM) was first postulated by Macklin and Macklin in 1944 [4]. The “Macklin effect” states that alveolar rupture will occur if a sufficiently large pressure gradient is generated against a closed glottis. At times, the emphysema extends to the epidural space [5]. There are several classifications of mediastinal emphysema, but the following five classifications by Engelsing [6] are easy to understand. Cases 1 and 2 are considered secondary mediastinal emphysema.

The occurrence of subcutaneous and mediastinal emphysema after lobectomy (or other lung operations) have been reported only rarely [1,7]. They seemed to be caused by minor air leakage – or perhaps rapid re-expansion- of the postoperative residual lung; however, such cases have not been reported yet, and this assumption is only based on clinical observations.

Given the rupture of the mediastinal pleura caused by mediastinal lymph node dissection, minor air leakage easily flows into the mediastinum. The high air flow from the lungs into the mediastinum further separates the air into low-resistance areas, including the subcutaneous space, leading to subcutaneous emphysema [8].

In the absence of complication, such as pneumothorax, conservative management is usually attempted, such as that observed in Case 1 [1]. However, it is uncommon that the mediastinal emphysema due to a minor air leakage will lead to a contralateral pneumothorax at 7 days postoperatively, as seen in “Case 2”; such complications have not been reported yet.

Generally, a pneumothorax of the residual lung on the surgical side occurs earlier than a pneumothorax on the contralateral lung. The earlier onset on the contralateral side in Case 2 suggests that the pressure required to rupture the contralateral mediastinal pleura and/or alveolus was smaller than the pressure required to deflate the residual lung on the surgical side. Such a pressure differential could arise if postoperative inflammatory pleural adhesions are particularly dense. In Case 2, minor air leakage would easily rupture a contralateral alveolus because the patient had advanced emphysema, which could worsen over several hours. Alternatively, it may have simply ruptured the mediastinal pleura. The mechanism is similar to that of bilateral pneumothorax [9], where the rupture of the alveoli within the lung parenchyma leads to the retrograde dissection of air along the bronchovascular sheaths of the bronchi and pulmonary vessels.

Another possibility is that the event was not a lobectomy-related complication at all, but rather a secondary spontaneous pneumothorax arising from pre-existing emphysematous disease in the contralateral lung. Forceful coughing can precipitate such a pneumothorax in emphysematous lungs [10].

In the case of a 2-year-old girl who developed delayed mediastinal and subcutaneous emphysema after extubation, which progressed to bilateral pneumothorax 8 h later [11], pneumothorax can develop and worsen several hours after the onset of mediastinal and subcutaneous emphysema. Therefore, closely monitoring the changes in the patient's condition is necessary.

The presence of subcutaneous and mediastinal emphysema immediately after surgery make radiographic interpretation difficult, and clinicians tend to focus on the surgical lung side to monitor for the development of a pneumothorax. If no pneumothorax is observed on the surgical side, conservative management will be performed. We recommend that pulmonologists should also monitor the contralateral side and that follow-up radiographic examinations should be performed a few hours later.

## 4 Conclusion

Herein, we present two cases of subcutaneous and mediastinal emphysema with different courses after lobectomy. Pulmonologists should monitor the side contralateral to the surgical site, even if the emphysema is caused by a postoperative minor air leakage.

## Author contribution

YS, HI, and TM: conceptualization, investigation, writing – original draft, and review and editing; KS, NH and TK: review and editing and supervision. All authors have approved the submitted version of the manuscript and agreed to be accountable for any part of the work.

## Informed consent

Written informed consent was obtained from the patient for publication of this case report and accompanying images. A copy of the written consent is available for review by the Editor-in-Chief of this journal on request.

## Ethical approval

Case reports are exempt from the “Ethical Guidelines for Life Sciences and Medical Research Involving Human Subjects” and do not require approval by the IRB of our institution.

## Guarantor

Dr. Kiyoshi Sato.

## Research registration number

This case report is not a ‘First in Man’ study and therefore was not required to be registered.

## Declaration of Generative AI and AI-assisted technologies in the writing process

During the preparation of this work, the authors used ChatGPT (OpenAI, GPT-4, April 2025 version) to improve the clarity and grammar of the manuscript. After using this tool, the authors reviewed and edited the content as needed and take full responsibility for the content of the publication.

## Funding

This research did not receive any specific grant from funding agencies in the public, commercial, or not-for-profit sectors.

## Conflict of interest statement

The authors declare that they have no known competing financial interests or personal relationships that could have appeared to influence the work reported in this paper.

## Data Availability

No data was used for the research described in the article.
